# Full-Self-Powered Humidity Sensor Based on Electrochemical Aluminum–Water Reaction

**DOI:** 10.3390/s21103486

**Published:** 2021-05-17

**Authors:** Marko V. Bošković, Biljana Šljukić, Dana Vasiljević Radović, Katarina Radulović, Milena Rašljić Rafajilović, Miloš Frantlović, Milija Sarajlić

**Affiliations:** 1 Department of Microelectronic Technologies, Institute of Chemistry, Technology, and Metallurgy, University of Belgrade, Njegoševa 12, 11000 Belgrade, Serbia; dana@nanosys.ihtm.bg.ac.rs (D.V.R.); kacar@nanosys.ihtm.bg.ac.rs (K.R.); milena@nanosys.ihtm.bg.ac.rs (M.R.R.); frant@nanosys.ihtm.bg.ac.rs (M.F.); 2 Faculty of Physical Chemistry, University of Belgrade, Studentski Trg 12-16, 11158 Belgrade, Serbia; biljka@ffh.bg.ac.rs; 3 CeFEMA, Instituto Superior Téchnico, Universidade de Lisboa, 1049-001 Lisbon, Portugal

**Keywords:** full-self-powered, breath monitoring, energy harvesting, humidity sensing, interdigitated capacitor, aluminum–air reaction

## Abstract

A detailed examination of the principle of operation behind the functioning of the full-self-powered humidity sensor is presented. The sensor has been realized as a structure consisting of an interdigitated capacitor with aluminum thin-film digits. In this work, the details of its fabrication and activation are described in detail. The performed XRD, FTIR, SEM, AFM, and EIS analyses, as well as noise measurements, revealed that the dominant process of electricity generation is the electrochemical reaction between the sensor’s aluminum electrodes and the water from humid air in the presence of oxygen, which was the main goal of this work. The response of the sensor to human breath is also presented as a demonstration of its possible practical application.

## 1. Introduction

Humidity sensing is of great importance in a wide variety of industrial processes, as well as in food production, health monitoring, and environmental protection [1]. Humidity monitoring is present in many fields, such as the pharmaceutical and chemical industry, microelectronics, agriculture, weather forecasting, as well as in daily life [1]. It is well known that the content of water vapor influences various physical, chemical, and biological processes [1]. Depending on the area of application, humidity is expressed in various ways. The most common ones are relative humidity (RH), absolute humidity, and dew point (DP) [1]. Various fields of application as well as the type of measurement unit (RH, DP) have required researchers to develop various types of humidity sensors [2,3,4]. In addition to the general requirements (linearity, reproducibility, accuracy, stability), the trend today is moving toward the development of sensors that can operate without relying on an external power supply, i.e., self-powered sensors [5]. The term “self-powered” typically refers to a system consisting of a sensor and an additional device used to harvest the available energy from the environment, thus providing the energy for the sensor operation [6]. Such devices harvest energy from motion, vibrations, light, electromagnetic radiation, fluid or air flow, and temperature gradients [7,8,9,10,11,12,13,14,15]. The selection between different available energies that could be harvested is made based on their abundance and availability in time.

There is an emerging field of chemical self-powered sensors which can function without even needing an energy-harvesting device. Instead, such sensors harvest energy in the process of sensing. Practically, the electrodes of the sensor are used as the sensing device and, at the same time, as the power source. Here, we call these “full-self-powered sensors”.

In our previous work, we developed a full-self-powered humidity sensor for breath humidity detection based on electrochemical interaction between aluminum electrodes and water vapor [16]. Given the importance of breath examination in modern medicine, this work offered a promising device for self-powered breath testing [17,18]. The sensor showed relatively high output signal, up to 1.5 V, very low noise, below 1 mV, an extremely fast response (rise time below 10 ms), and it was tested for applications in human breath detection. The sensor was designed as an interdigitated capacitor (IDC) made of a thin aluminum film. The proposed sensing principle of the sensor is based on the reaction between aluminum and oxygen dissolved in water on its surface, similar to aluminum–air batteries [16,19,20]. In this report, we investigate in detail the principle of operation of the aluminum IDC sensor, especially looking for evidence of an aluminum–water electrochemical interaction. The full understanding of the underlying process required micro- and nanotesting of the sensor’s surface using the following methods: micro-Fourier transform infrared spectroscopy (µ-FTIR), atomic force microscopy (AFM), scanning electron microscopy (SEM), energy-dispersive X-ray spectroscopy (EDS), electrochemical impedance spectroscopy (EIS), and X-ray diffraction (XRD) [21]. We also considered possible concurrent processes such as the hydrovoltaic effect [22,23] and radiofrequency harvesting [12].

## 2. Materials and Methods

### 2.1. Fabrication

**Figure 1:**
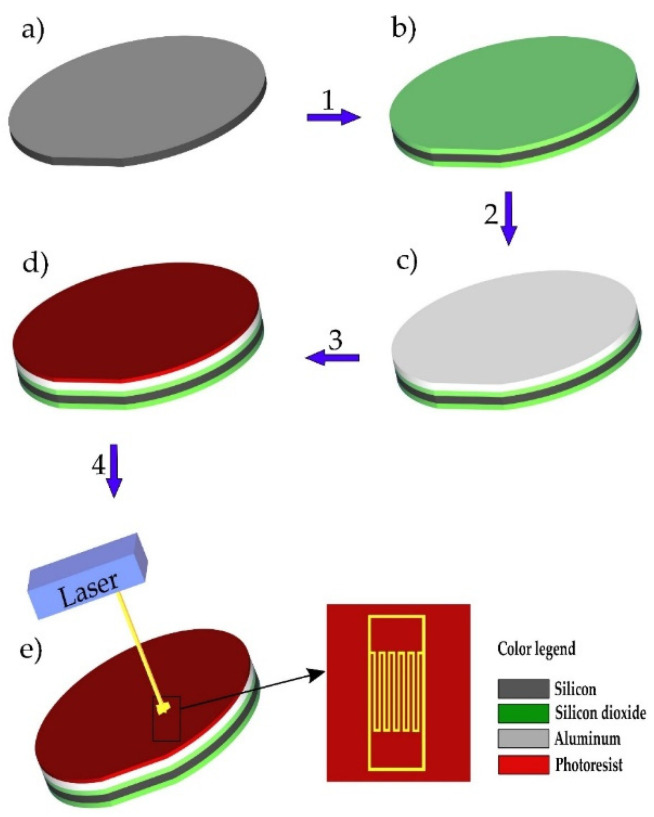
Depiction of the fabrication procedure: (**a**) Starting wafer; (**b**) thermal oxidation of the silicon wafer; (**c**) sputtering of aluminum 1% silicon; (**d**) spin coating of the photoresist; (**e**) exposure to laser light.

**Figure 2:**
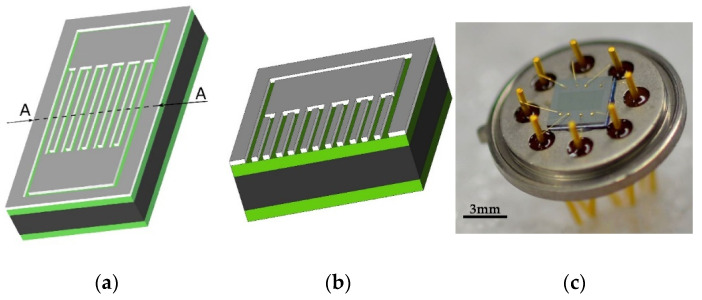
(**a**) 3D design of the single sensor battery; (**b**) cross-section through A–A line; (**c**) photograph of the finished sensor on a TO-8 housing. Scale bar 3 mm.

The schematic of the sensor fabrication procedure is given in Figure 1. The starting material was a 3″ silicon wafer, <100> orientation, n-type, 380 µm thick, as seen in Figure 1a. The first step was thermal oxidation at 1100 °C for 105 min, which led to the formation of 0.6 µm thick silicon dioxide on both sides of the wafer, as seen in Figure 1b. The oxide layer was used to electrically insulate the structure from the silicon substrate. Afterwards, a thin film of aluminum 1% silicon was deposited by DC magnetron sputtering (Sputtersphere 822, Material Research Corporation, Orangeburg, NY, USA), as seen in Figure 1c. The next step was the spin coating of photoresist (AZ-1505), as seen Figure 1d, which was followed by exposure to the laser light which followed the designed pattern, as seen in Figure 1e (Laser Writer LW405 MicroTech, Palermo, Italy). Finally, wet chemical etching of the exposed area was performed, with an aqueous solution that contained a mixture of 5% nitric acid, 75% of phosphoric acid, and 10% of acetic acid. The wafer was diced on a dicing machine (Dicing saw 602M, GS MicroAutomation, Sunnyvale, CA, USA), thus producing chips of a 3.6 mm × 3.6 mm size. The image of the obtained structure and its cross-section are presented in Figure 2a,b. Finally, the fabricated sensor chips were glued to a TO-8 housing made of Kovar (Nickel-Cobalt-Ferrous alloy) using the Scotch White Glue, as seen in Figure 2c.

### 2.2. Scanning Electron Microscopy

**Figure 3:**
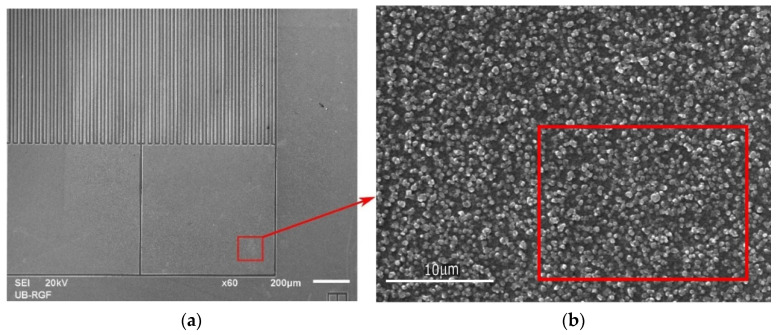
(**a**) SEM image of the sensor, scale bar 200 µm. (**b**) SEM image, scale bar 10 µm, with a marked area where EDS analysis was performed.

Figure 3 presents a SEM image of the sensor as seen on two different magnifications (a,b). Elemental analysis of the sensor surface was obtained through energy-dispersive X-ray spectroscopy (EDS). It was found that aluminum, silicon, and oxygen are present at a volume of 61.72%, 26.56%, and 11.72%, respectively (Table 1). Scanning electron micrographs were obtained using JSM-6610LV (JEOL, Tokyo, Japan).

### 2.3. Atomic Force Microscopy

**Figure 4:**
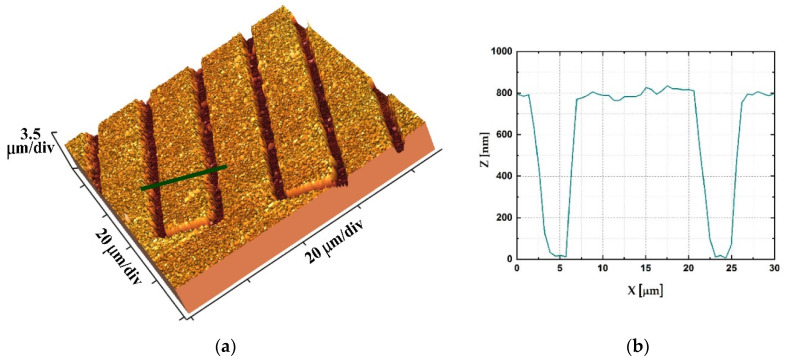
(**a**) 3D AFM image of the sensor detail; (**b**) profile obtained via AFM along the line marked as green in (**a**).

Additional characterization of the sensor’s surface was performed using atomic force microscopy. The measurements were performed using Thermomicroscopes AutoProbe CP (Veeco, Munich, Germany). Figure 4 presents a 3D image of the sensor detail (a) and the corresponding profile (b). As can be seen from Figure 4b, the thickness of the sputtered material is 800 nm. The width of digits is 14 µm, while the clearance between digits is 6 µm. The average roughness is 40 nm, which gives a specific surface area which is 10% larger than the geometrical one. Analysis was performed using Image Processing and Data Analysis Software (TM microscopes, Version 2.1.15).

### 2.4. X-ray Diffraction

**Figure 5:**
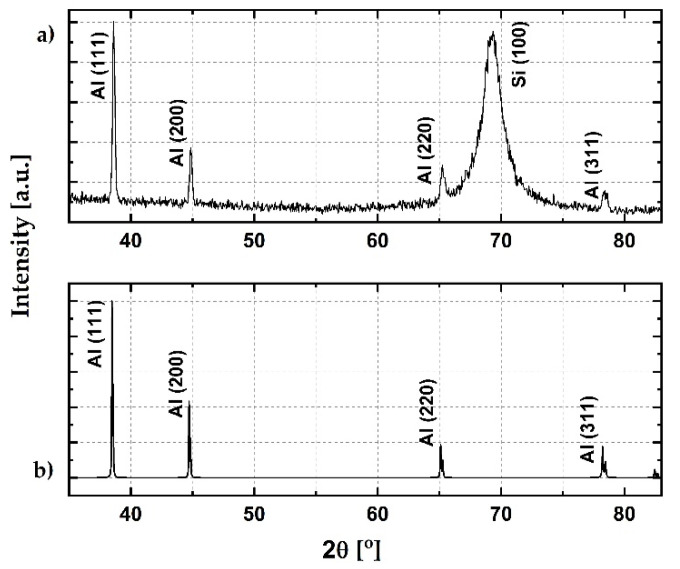
(**a**) Obtained XRD diffractogram; (**b**) theoretical diffractogram for aluminum.

Structural characterization of the sputtered material was conducted via XRD analysis. Measurements were performed on the Rigaku Ultima IV with Cu target (Kα radiation, λ = 0.154178 nm) (Rigaku, Tokyo, Japan). The theoretical pattern was calculated using the VESTA software [24]. As input for the calculation, a face-centered cubic cell with a 404 pm lattice constant was used [25]. The experimental pattern is presented in Figure 5a, together with the theoretical diffractogram in Figure 5b. The peak at 2θ of 38.6° originates from the reflection from the Al (111) crystalline plane. The lower-intensity peak at 2θ of 44.9° originates from the reflection from the Al (200) plane. There are two low-intensity peaks at 2θ of 65.3° and 78.3° which originate from the reflections from the Al (220) and Al (311) planes, respectively [26,27]. The high-intensity peak at 2θ of 69.4° originates from the reflection from the Si (100) plane [28]. By comparing peak intensities from theoretical and experimental diffractograms, it can be concluded that the (111), (200), and (220) planes are equally present, while the (311) plane is 50% less abundant. The crystallinity index was calculated at a value of 0.85.

### 2.5. Electrode Activation 

**Figure 6:**
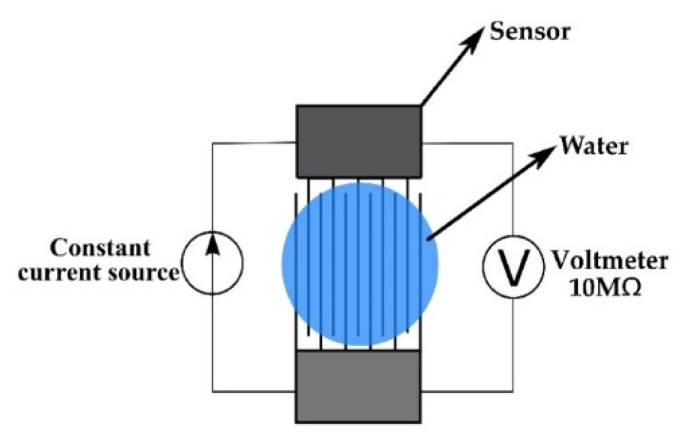
Electrode activation circuit.

**Figure 7:**
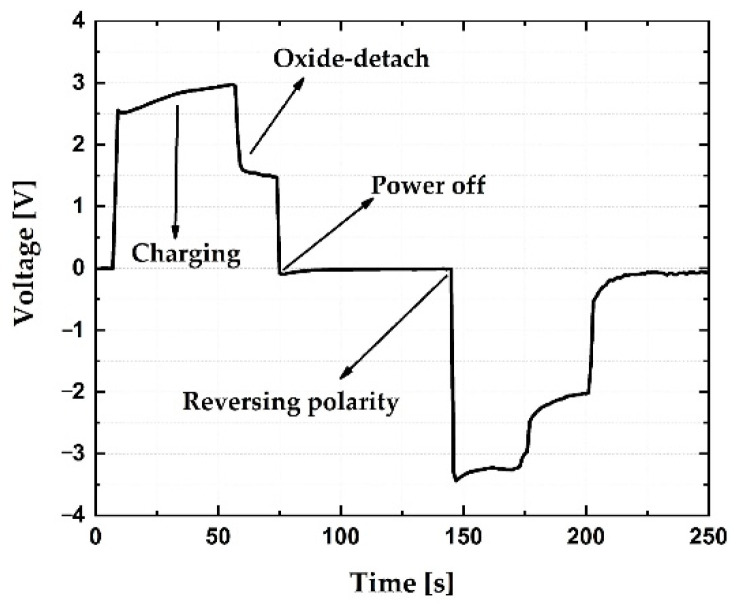
Time dependence of voltage during the electrode activation process.

In order to make the sensor sensitive to moisture, the activation procedure must be performed [16]. Before activation, the sensor was providing no output when subjected to high water vapor concentration. It is assumed that this inactivity is due to the coverage of the sensor surface with aluminum oxide. The oxide was formed during the sputtering process, or through spontaneous formation in the ambient atmosphere [29,30]. A schematic of the electrode activation process is given in Figure 6. The sensor is connected to a constant current source, and a current of 1 µA is applied. A digital voltmeter is used to measure the voltage drop on the sensor. A droplet of demineralized water was dropped on the sensor surface, where it acts as an electrolyte. A typical diagram for the electrode activation is given in Figure 7. During the activation process, three phases can be distinguished. After applying the current, charge accumulation on the electrode surface occurs, which manifests as a voltage increase. As a result, the electric field is created between the electrode and the electrolyte. At a certain moment, the electrostatic force which acts on the surface oxide overcomes adhesive forces, and oxide detaches from the electrode surface. As soon as the insulating layer is removed, current can flow into the water which is now undergoing electrolysis. This process is seen as a voltage drop in Figure 7, which indicates that the activation procedure is complete. When the current source is turned off, the voltage decays. The same procedure must be performed with reversed polarity in order to remove the oxide layer from the other electrode.

## 3. Results and Discussion 

### 3.1. Human Breath Test

**Figure 8:**
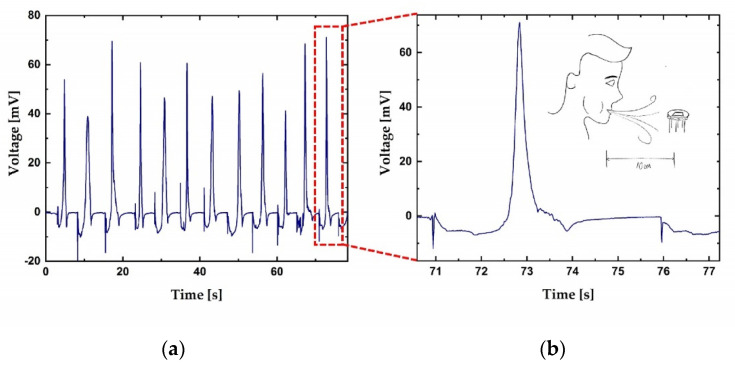
(**a**) Sensor’s response to periodical human exhalation from a 10 cm distance; (**b**) response to a single breath blow.

After the activation process, the sensor becomes sensitive to the surrounding humidity, for instance, humidity from human breath. Figure 8a shows the response of the sensor to breath blow in the time span of approximately 80 s. In Figure 8b, a zoom into a single breath blow event is given. The sensor was exposed to a direct breath blow from a person blowing from a distance of about 10 cm. The volunteer blew on the sensor’s surface in short bursts, with a repetition period of about 10 s. The signal climbed to 70 mV, while its polarity was changeable. The response was fast, with a rise time in the range of 100 ms. The sensor was connected to a voltmeter with shielded cables. The input impedance of the voltmeter was set to 10 MΩ, with 1PLC (PLC = power line cycle; 1 PLC means averaging of the acquired signal in 20 ms). The experiment shows that the sensor reacts to high humidity levels present on its surface. Thus, it could be used as a breath detection device.

### 3.2. Electrochemistry of Aluminum–Water Reaction 

The behavior seen in Figure 8 can be described as an electrochemical reaction similar to that in an aluminum–air battery [31]:

Cathode: O_2_ + 2H_2_O + 4eˉ −> 4OHˉ (1)

Anode: Al −> Al^+3^ + 3eˉ (2)

Total: 4Al + 6H_2_O + 3O_2_ −> 4Al(OH)_3_ (3)

The anodic half-reaction has a theoretical potential of −2.31 V, while the cathodic half-reaction has a potential of +0.4 V (versus normal hydrogen electrode) [19,32]. Thus, the theoretical value for the aluminum–air battery is expected to be 2.71 V. In practice, the open-circuit voltage of these batteries is significantly lower, and its value is between 1.2 V and 1.6 V [31]. The discrepancy between the theoretical and the practical open-circuit voltages is a consequence of various unfavorable reactions, which lead to the passivation of the aluminum surface [31,33]. The considered sensor has a lower voltage than the aluminum–air battery due to its electrolyte conductivity. Namely, the experimental voltage of approximately 1.6 V for aluminum–air batteries is accomplished using strong electrolytes, such as NaOH, NaCl, and KOH [34]. In the presented experiment with human breath, the adsorbed water has relatively weak electrolytic conductance in comparison to the mentioned electrolytes, thus leading to a significant voltage drop.

The random change of the output voltage polarity can be explained by the following consideration. The sensor is fabricated with both electrodes made of the same material, so the cathode and the anode are not predefined by design. Which electrode will be the cathode and which the anode depends on the oxygen concentration on its surface, i.e., on the amount of adsorbed water. Since this is a statistical process, it appears that the electrodes may swap roles as anode and cathode over time, which will manifest in the instability in the output voltage polarity.

### 3.3. Micro-Fourier Transform Infrared Spectroscopy

**Figure 9:**
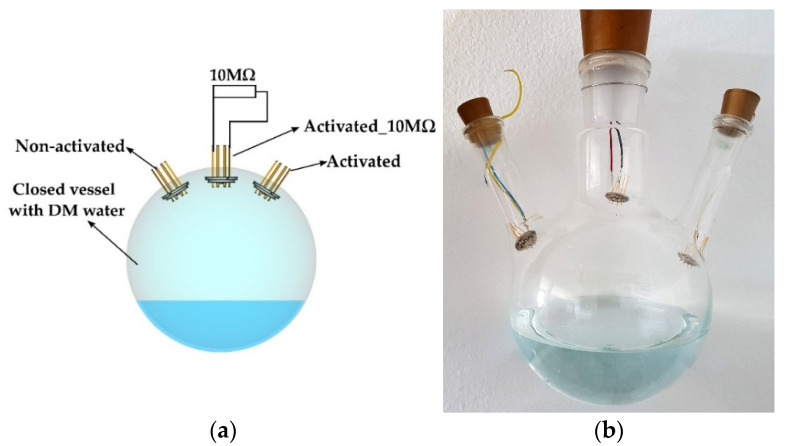
(**a**) A graphical illustration and (**b**) photograph of sensor preparation for µ-FTIR measurement.

**Figure 10:**
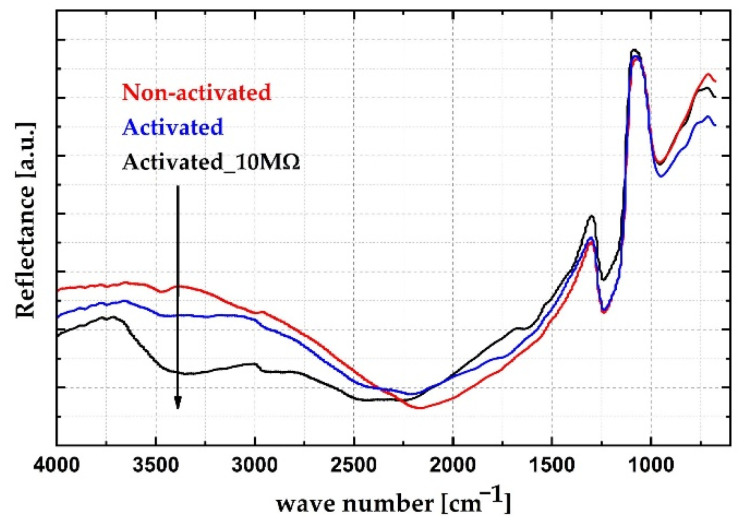
FTIR spectra of the tested samples: red—non-activated sensor; blue—activated sensor with open ends; black—activated sensor connected to a 10 MΩ resistor.

In cases where the sensing mechanism of the sensor is based on the operating principle of the aluminum–air battery, it was expected that aluminum–hydroxide would appear as a reaction product (3). In order to verify this assumption, the sensor surface was characterized by micro-Fourier transform infrared spectroscopy. Reflectance spectra were acquired using an iN-10 Infrared Microscope (Thermo Fisher Scientific, Waltham, MA, USA) with a wavenumber range from 675 cm^−1^ to 4000 cm^−1^. Three sensors were investigated, of which two were activated, and one was not. The activated sensors were kept in a closed vessel (three-neck round-bottom glass flask) half-filled with demineralized water, so that sensors were constantly at 100% RH for 10 days, in order to form reaction products to be characterized by µ-FTIR. One of the activated sensors was connected to a 10 MΩ resistor so that the current through the sensor was always present. The other activated sensor was kept with the ends open, i.e., no current flowing. The non-activated sensor was kept under the same conditions to make the results of the characterization comparable. A graphical illustration and a photograph of the preparation process are presented in Figure 9a,b, respectively. All sensors were dried using a nitrogen gun before measurement. µ-FTIR measurements were performed with the illuminated surface of 400 × 400 µm^2^ positioned on the sensor digits. The obtained µ-FTIR spectra are given in Figure 10. The µ-FTIR spectra showed that aluminum oxide is present in all measured samples. The absorption band at 1250 cm^−1^ corresponds to Si–O stretching vibration [35,36]. The broad absorption band at 950 cm^−1^ is attributed to the Al–O stretching vibration [37]. The broad band at 3400 cm^−1^ corresponds to various aluminum-based hydroxyls [37,38,39,40,41]. The sample which was not activated had no electrochemical processes on its surface, which corresponds to the relatively higher signal around 3400 cm^−1^. The samples which were activated produced Al(OH)_3_ on their surfaces. Consequently, the signal around 3400 cm^−1^ is significantly lower, which confirms the hypothesis of the electrochemical interaction between aluminum and water in the presence of oxygen (1–3). This observation shows that aluminum hydroxyls are formed as products of the sensing process, which indicates that the principle of operation of the sensor is based on the electrochemical aluminum–water reaction, as seen in Figure 10.

### 3.4. Electrochemical Impedance Spectroscopy

**Figure 11:**
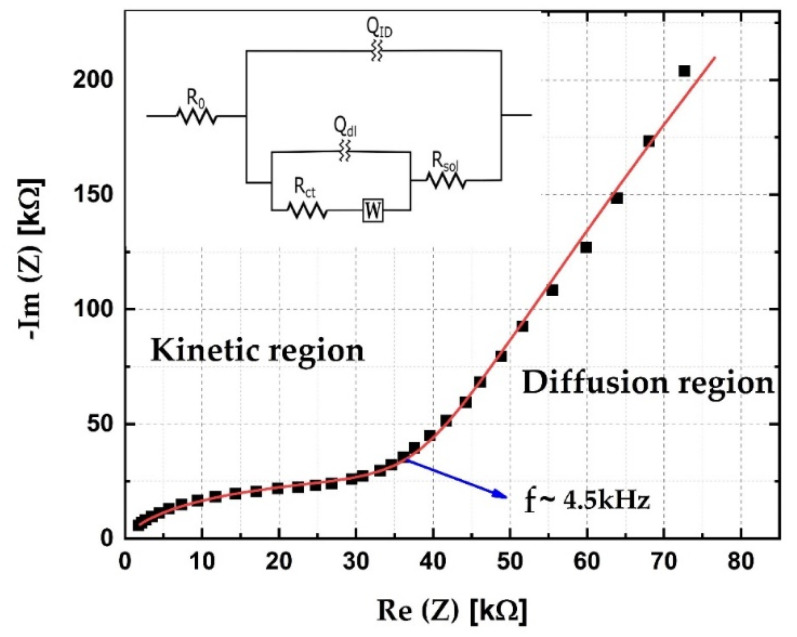
Nyquist plot of experimental (symbols) and fitted results (line) with equivalent circuit (inset).

The electrochemical characterization of the sensor was conducted via electrochemical impedance spectroscopy. Measurements were obtained using the Ivium Vertex 46804 (Ivium, The Netherlands) in a frequency range from 300 Hz to 200 kHz, with the shorted reference electrode and counter electrode, at the open circuit potential. In order to make the electrochemical reaction possible, a droplet of demineralized water was placed on the sensor’s surface. A Nyquist diagram is presented in Figure 11, together with the fitted data. The equivalent circuit which was used for analyzing the impedance data is given as an inset in Figure 11. Capacitive contributions were analyzed as constant phase elements (CPE), whose impedance is defined as:

(4) $$Z{\left(j\omega \right)}_{CPE}=\frac{1}{{\left(j\cdot \omega \right)}^{n}\cdot Q}$$

where ω is the angular frequency, *Q* is the admittance for ω=1, and *n* is the depression parameter, with values between −1 and 1. When *n* = 1, CPE represents a perfect capacitor, and when *n* = −1, it represents a perfect inductor [42]. For capacitance modeling, values of *n* are typically between 0.8 and 1 [43]. Constituent parts of the equivalent circuit are: R_0_ is the resistance of the cables, R_ct_ is the charge transfer resistance, R_sol_ is the electrolyte resistance, Q_ID_ is the interdigitated (geometrical) capacitance, Q_dl_ is the double-layer capacitance, and W is the Warburg element for modeling diffusion behavior. Values of imaginary and real components of the equivalent circuit are presented in Table 2. The depression parameter (*n*) has a value of approximately 0.9 for the double-layer capacitance, as well as for the geometrical capacitance. This deviation from the behavior of the ideal capacitor is most likely a consequence of the surface roughness [44]. The roughness of the sensor’s surface was confirmed via AFM measurements (Figure 4).

### 3.5. Possible Concurrent Processes 

In addition to the considered electrochemical reaction, the sensor could be influenced by radiofrequency (RF) and electromagnetic (EM) interferences. These interferences may manifest as a reading offset or may result in noise and erratic readings. Additionally, the hydrovoltaic effect might come into play and increase the voltage level, apart from RF interferences [22,23]. A set of experiments was conducted in order to analyze the contribution of possible concurrent processes on the sensor’s output.

#### 3.5.1. RF Interference

**Figure 12:**
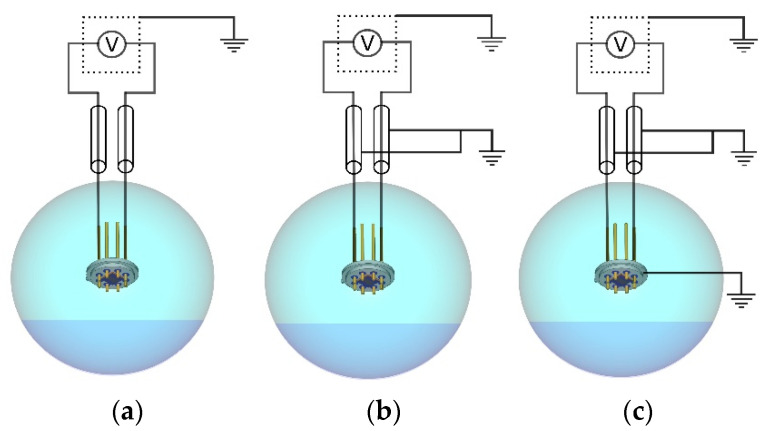
Measurement configurations: (**a**) voltmeter is grounded; (**b**) voltmeter and cable shields are grounded; (**c**) voltmeter, housing, and cable shields are grounded.

**Figure 13:**
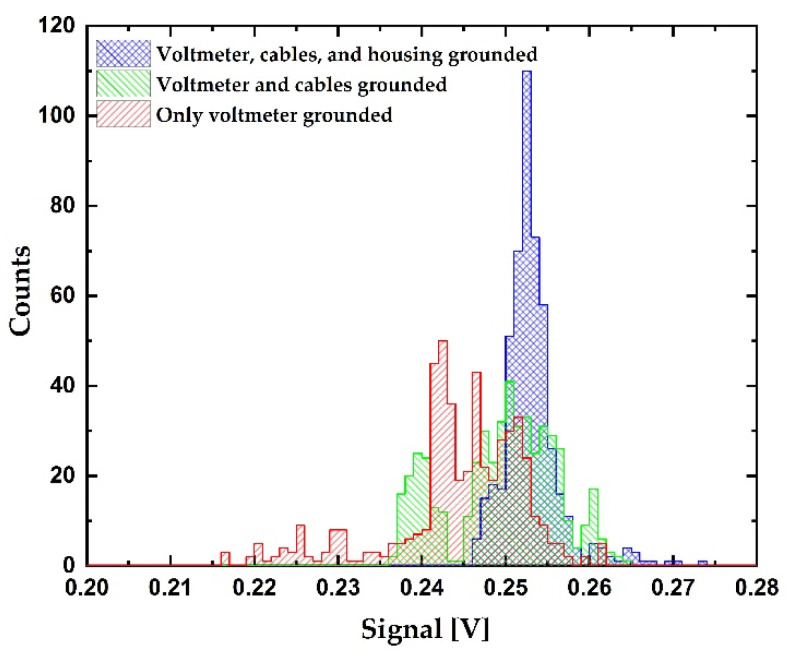
Histogram of the signal: red—voltmeter is grounded; green—voltmeter and cable shields are grounded; blue—voltmeter, shields, and housing are grounded. Number of events is 501 in all cases. Bin size is 1 mV.

The level of noise at the sensor’s output was assessed by systematically connecting the components of the system to the ground. Measurements were performed in three different configurations, as seen in Figure 12. In the first configuration, the sensor was placed in a closed vessel with 100% RH atmosphere and connected to a grounded voltmeter with shielded cables, as seen in Figure 12a. In the second configuration, cable shields were also grounded, as seen in Figure 12b. In the third configuration, the sensor housing, the cable shields, and voltmeter were all grounded, as seen in Figure 12c. All the measurements were performed at 25 °C, while relative humidity was monitored using the reference sensor (Honeywell HIH 4001). The input impedance of the voltmeter was set to 10 MΩ with 10 PLC (10 PLC means averaging of the acquired signal in 200 ms). Distribution curves and standard deviation (STD) values of the measured signals are presented in Figure 13 and Table 3, respectively. The number of events was 501 in all measurements. The bin size was 1 mV. The signal-to-noise ratio (SNR) was calculated as mean/STD. It is presented in units of dB (20 × log (Mean/STD)). The obtained results show that additional grounding of the components significantly reduces the noise in the system. When all components are grounded, the standard deviation was decreased by more than 50% in comparison to that without grounding. Even the highest standard deviation gives the signal-to-noise ratio of 30 dB, which is still an acceptable value for this purpose. This shows that the influence of RF interference is relatively low, and it can be further diminished by the proper grounding of cables, sensor housing, and the voltmeter.

In the case where the sensor was exposed to a dry atmosphere, much lower voltages should be obtained. For this purpose, the sensor was placed in the glass bottle, under constant flow of nitrogen to provide a water-free atmosphere. In this manner, the measured voltage is a consequence of RF harvesting in the cables and noise in the voltmeter. The mean value of the measured signal was 80 µV. This is much lower than in the case of the sensor in the wet atmosphere, meaning that the contribution from RF harvesting in the cables and the noise in the voltmeter is 0.03%, which is negligible. 

#### 3.5.2. Hydrovoltaics

Another mechanism for energy harvesting from the interaction between solid surface and water is the hydrovoltaic effect [22,23,45]. Devices whose working mechanism is based on the hydrovoltaic principle generate electricity if at least one of the following conditions is fulfilled: the presence of diffusion as a consequence of concentration gradient, fluid flow (waving, streaming, pressure or gradient-induced), or gradient in ion concentration as a consequence of nonequilibrium in water desorption from its surface [23]. Since none of these conditions were met, the hydrovoltaic effect can be ruled out as a concurrent process.

## 4. Conclusions

The principle of operation of the full-self-powered sensor was investigated. The signal measurements in various experimental set-ups, as well as theoretical consideration, showed that the main contribution to the sensor’s signal originated from an electrochemical aluminum–water reaction. Concurrent processes such as RF harvesting and hydrovoltaic can be ruled out as the dominant source of the signal. The atomic composition, surface morphology, and geometric profile of the obtained structure were investigated using microscopy analysis (SEM and AFM). SEM analysis showed that sensor digits have a grainy structure. EDS analysis of the digits revealed the element composition to contain aluminum, silicon, and oxygen. AFM measurements revealed the geometrical profile of the obtained structure, together with its surface roughness. XRD patterns revealed that the sputtered material has a highly crystalline structure. The electrode activation procedure was discussed together with the sensor’s sensing mechanism. FTIR spectra showed that aluminum hydroxyls are produced in the sensing reaction. EIS measurement revealed the equivalent circuit of the sensor as an electrochemical system, thus confirming the previously anticipated schematic and giving exact parameter values for the circuitry components. The sensor’s response to high relative humidity was tested through the human breath experiment. The signal of the sensor on breath humidity testing was in the range of 100 mV and with very low noise, and thus easily measurable by standard instruments. Since both electrodes are made of aluminum, both of them can react to moisture in the sensing process. This triggers a flip in the polarity, which was observed in the experiments. The signal polarity flip will be the subject of our future work. Addressing the selectivity and stability parameters along with the sensitivity level will also be the subject of our future work.

## Figures and Tables

**Table 1:** Atomic composition of the sensor surface.

Element	Al	Si	O	Total
Atomic %	61.72	26.56	11.72	100

**Table 2:** Values for imaginary and real parameters of equivalent circuit.

Q_id_ [Ω^−1^cm^−2^s^0.92^]	n_id_	Q_dl_ [Ω^−1^cm^−2^s^0.9^]	n_dl_	W [Ω^−1^s^0.5^]	R_0_ [Ω]	R_ct_ [Ωcm^2^]	R_sol_ [Ωcm^2^]
13 × 10^−9^	0.92	757 × 10^−9^	0.9	1.96 × 10^−6^	0.9	165	162

**Table 3:** Standard deviation, mean, and signal-to-noise ratio of the three considered configurations.

	STD [V]	Mean [V]	SNR [dB]
Voltmeter, cables, and housing grounded	3.4 × 10^−3^	0.253	37.4
Voltmeter and cables grounded	6.5 × 10^−3^	0.250	31.7
Voltmeter grounded	8.1 × 10^−3^	0.255	30.0

## Data Availability

The data is available at Mendeley Data, DOI:10.17632/rjm3gknm27.1.
